# Diagnosis of Spontaneous Massive Fetomaternal Hemorrhage: A Case Report and Literature Review

**DOI:** 10.1002/jcu.24062

**Published:** 2025-05-08

**Authors:** Martina Derme, Adele Vasta, Valentina Tibaldi, Paola Galoppi, Valentina D'Ambrosio, Daniele Di Mascio, Ilenia Mappa, Samia Ennaciri, Manuel Esposito, Valentina Demarco, Antonella Giancotti, Giuseppe Rizzo

**Affiliations:** ^1^ Azienda Ospedaliero‐Universitaria Policlinico Umberto I Rome Italy; ^2^ Department of Maternal and Child Health and Urological Sciences Sapienza University of Rome Rome Italy; ^3^ Department of Obstetrics and Gynecology Università di Roma Tor Vergata Rome Italy; ^4^ Department of Obstetrics and Gynecology AOUI Verona, University of Verona Verona Italy

**Keywords:** Doppler, fetal anemia, fetomaternal hemorrhage, fetomaternal transfusion, FMH, middle cerebral artery, obstetrics

## Abstract

Fetomaternal hemorrhage (FMH) is the passage of fetal blood through the maternal circulatory system before or during delivery. Due to the nonspecificity of clinical manifestations, FMH is rarely diagnosed. When it occurs rapidly, it could have catastrophic consequences. Decreased fetal movements and a sinusoidal cardiotocographic (CTG) for more than 30 min are characteristic findings. This paper presents a case report of a 34‐year‐old woman at 38 + 4 weeks with reduced fetal movements and a sinusoidal CTG. An emergency cesarean section was performed, delivering a pale newborn with a hemoglobin of 4.3 g/dL. A comprehensive review of the available literature is then reported.

## Graphical abstract

Fetomaternal hemorrhage (FMH) is the passage of fetal blood through the circulatory system of the mother either before or during delivery, and it is rarely diagnosed prenatally. This paper aims to present a comprehensive review of the literature allowing us to offer comprehensive insights into the diagnosis and management of FMH.

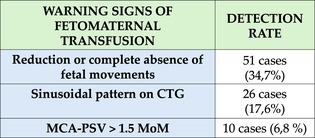

## 1 Introduction

The incidence of spontaneous massive fetomaternal hemorrhage (FMH) is difficult to establish because it is rare, unpredictable, and the signs and symptoms are neither specific nor reliably present (Sebring and Polesky 1990; Troìa et al. 2019). It may occur before delivery or in labor, with an acute onset, usually associated with worse neonatal outcomes, or chronic, giving the fetus the opportunity to compensate through increased red blood cell production. It is distinguished into massive or small depending on whether the fetal erythrocyte volume in maternal circulation is up to 150 mL, although it should be corrected for the total fetoplacental blood volume, which correlates with fetal size and gestational age (Kecskes 2003).

The most common presenting symptom of this condition is a reduction in perceived fetal movements (27%–54%) (Christensen et al. 2013). At cardiotocographic (CTG) assessment, it is common to find a continuous or intermittent sinusoidal pattern (Giacoia 1997). Research by Markham et al. (2006) suggested that at least 50% of fetal blood loss in the maternal circulation is required before signs of fetal impairment become evident. In patients in whom FMH is suspected, ultrasound assessment is useful, in particular, the study of peak systolic velocity in the middle cerebral artery. There is evidence that a value of peak systolic velocity greater than 1.5 MoM is indicative of severe anemia (Guo et al. 2011; Mari et al. 2000). Laboratory results showing increased production of red blood cell precursors and increased circulating reticulocyte counts in the fetus suggest that FMH occurred a day or two before birth (Nicolaides et al. 1988). The Kleihauer–Betke (KB) test is the main diagnostic test for confirming and quantifying the proportion of fetal hemoglobin (Hb) within the maternal circulation (Sebring and Polesky 1990; Kleihauer et al. 1957). We reported a case of idiopathic FMH in a pregnant woman who was admitted to our center at 38 weeks and 4 days of pregnancy for reduced fetal movements. We then performed a comprehensive review of the literature aimed at offering insights into the diagnosis and clinical management of FMH.

## 2 Case Report

**FIGURE 1:**
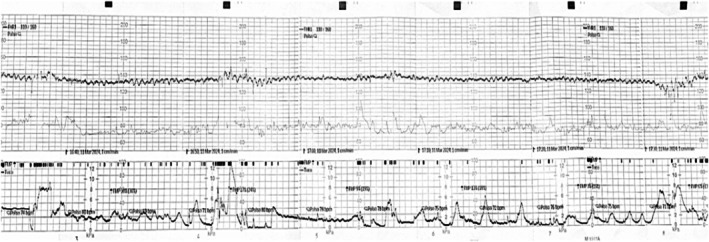
The nonstress test on admission with a variability of about 7 bpm, the presence of active fetal movements, the absence of acceleration and deceleration with an intermittent sinusoidal pattern for a time of 82 min, and irregular contractile activity.

The case hereby reported was of a 34‐year‐old, para 0, gravida 1 woman, with blood type A Rh‐positive, admitted to our department at 38 weeks and 4 days for referred reduced fetal movements for 6 h and presence of incoordinate contractile activity. No history of trauma was reported. She had an uncomplicated antenatal course during pregnancy with no personal history of abnormal bleeding, hypertension, or gestational diabetes. No invasive prenatal tests were performed on the basis of a low‐risk noninvasive prenatal test (NIPT). The last ultrasound examination performed at 35 weeks and 4 days showed a singleton fetus with an estimated fetal weight of 2350 g, normal amniotic fluid index (AFI), normal maternal and fetal Doppler findings, and a normal placenta in appearance and location. At admission, the maternal vital parameters were normal, with a heart rate of 83 bpm and a blood pressure of 96/49 mmHg. At the obstetrical examination of a long, closed, and posterior cervix, there were no signs of vaginal bleeding, intact amniotic membranes, and high station cephalic fetus. A nonstress test on admission demonstrated a baseline of 140 bpm with a variability of about 7 bpm, presence of active fetal movements, and absence of acceleration and deceleration with an intermittent sinusoidal pattern for a duration of 82 min with irregular contractile activity (Figure 1). No changes in fetal heart patterns occurred after conservative attempts of fetal resuscitation including maternal position change and intravenous fluid administration.

**FIGURE 2:**
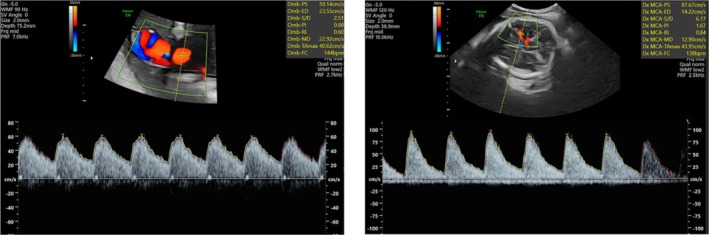
The fetal Doppler exam showing an umbilical arterial pulsatility index (UA‐PI) of 0.88, a middle cerebral artery pulsatility index (MCA‐PI) of 1.67, and a cerebroplacental ratio (CPR) of 1.89 (at 47° percentile). The middle cerebral artery peak systolic velocity (MCA‐PSV) was 87.67 cm/s, equal to 1.48 multiples of median (MoM). Although the image is technically suboptimal, characterized by low magnification and an insonation angle greater than 30°, it is important to underline that, in emergency situations, such limitations may be unavoidable. Notably, an insonation angle > 30° typically underestimates MCA‐PSV; yet in this case, the elevated value was still clearly diagnostic.

An ultrasonographic exam performed at admission showed a cephalic singleton fetus with a regular fetal heart rate (FHR) (135 bpm), a maximum amniotic fluid vertical pocket of 66.3 mm, and a normoinsert anterior placenta. In addition, a fetal Doppler examination was performed showing an umbilical arterial pulsatility index (UA‐PI) of 0.88, a middle cerebral artery pulsatility index (MCA‐PI) of 1.67, and a cerebroplacental ratio (CPR) of 1.89 (at 47° percentile) according to our reference limits for gestation (Rizzo et al. 2022). The middle cerebral artery–peak systolic velocity (MCA‐PSV) was 87.67 cm/s, equal to 1.48 multiples of median (MoM), which, for the gestational age, is suggestive of fetal anemia as shown in Figure 2.

**FIGURE 3:**
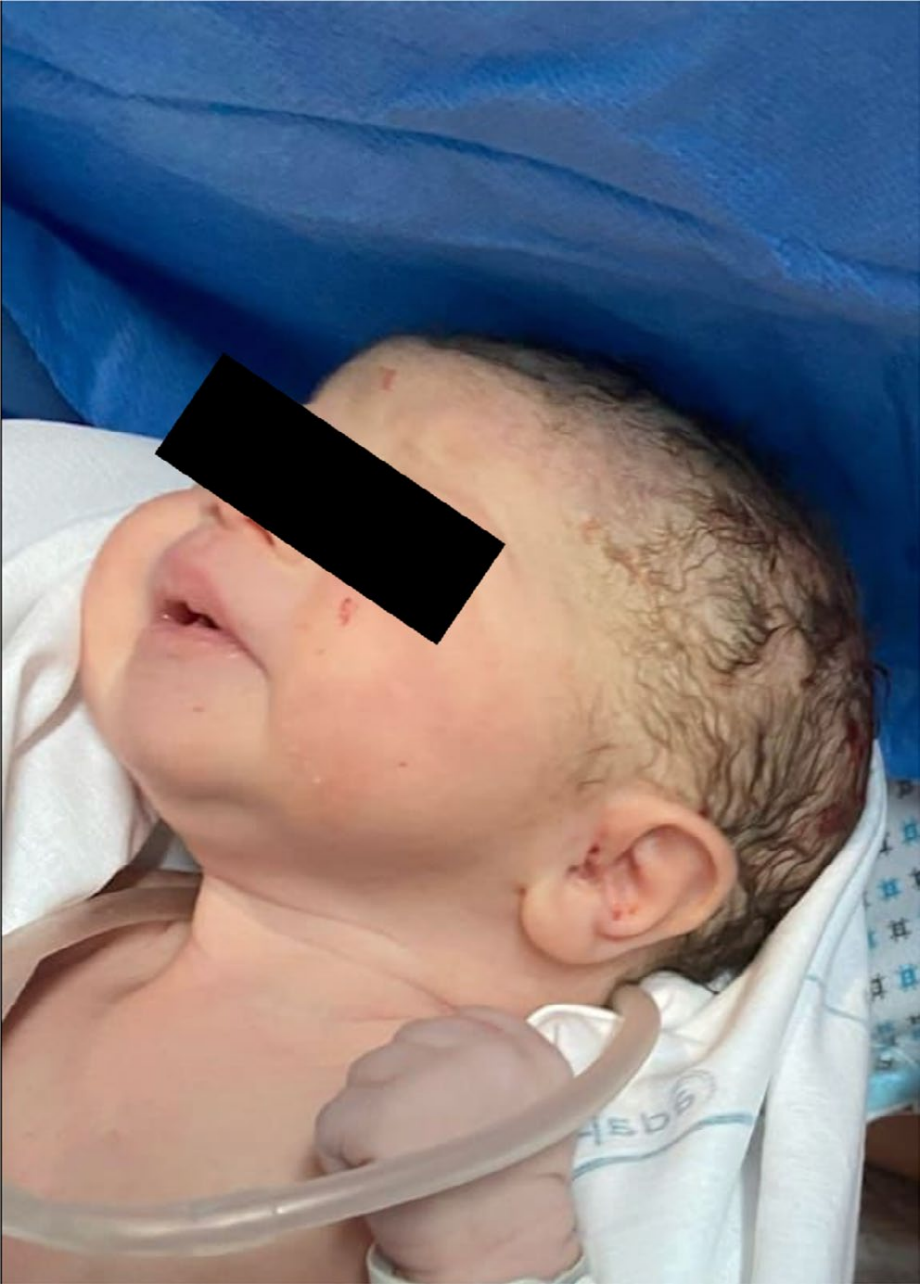
“Ghost‐like” appearance of the baby immediately after delivery.

Indeed, fetal Hb level estimated from this measurement was 10.2 g/dL according to the Fetal Medicine Foundation prediction of anemia algorithm (https://fetalmedicine.org/research/assess/anemia). The overall fetal biophysical profile (BPP) was therefore 8 out of 10: 2 points for fetal breathing movements (FBM), 2 points for fetal body movements, 2 points for fetal muscle tone, 2 points for amniotic fluid volume, and finally 0 points for FHR because of the sinusoidal pattern at the nonstress test. The woman was admitted to the labor ward, and the results of the blood tests performed showed no maternal pathological status of anemia (Hb 13.2 g/dL; platelet count [PLT] 181 × 10^9^/L; white blood cell [WBC] 8.02 × 10^9^/L). On the basis of the persistence of the sinusoidal CTG pattern, an emergency cesarean section was performed A female newborn weighing 2720 g was delivered with an Apgar score of 5 in the first minute that improved to 6 at 5 min. The clinical examinations showed a pale with “ghost‐like” appearance (as shown in Figure 3) with the following vital signs: Blood pressure was 60/40 mmHg, and heart rate was 145 beats/min. The umbilical artery gas analysis showed a pH of 7.44, with a base excess (BE) of—2.1 mmol/L, partial pressure of oxygen (pO_2_) of 51 mmHg, partial pressure of carbon dioxide (PCO_2_) 31 mmHg. After placing a nasal continuous positive airway pressure (NCPAP) to ensure continuous positive airway pressure, the infant was directly transferred to the neonatal intensive care unit (NICU) where an urgent pulmonary RX was performed. The vital signs were stable and no clinical signs of hepatosplenomegaly or of peripheral edemas were present. The first blood test collected from venous blood in the first hour of life showed a neonatal hemoglobin of 4.3 g/dL, hematocrit of 15.3%, leukocytes 22.70 × 10^9^/L, platelets 401 × 10^9^/L, reticulocytes 20.7% (262 × 10^9^/L), CHR (reticulocyte hemoglobin content) 34.4 pg. After 4 h, a transfusion with erythrocyte concentrate group O Rh‐negative was given. The transfused volume was 44 mL with a hematocrit of 76%, in 4 h at a velocity of 11 mL/h: with progressive improvement of vital parameters, a heart rate of 125 beats/min, blood pressure of 61/51 mmHg (mean arterial pressure 55 mmHg), Oxygen saturation (SpO_2_) 98%, fraction of inspired oxygen (FiO_2_) of 0.21 and a temperature of 36.4°C. The following day, the hemoglobin levels increased from 4.3 g/dL to 10.8 g/dL, and on Day 4 after blood transfusion, hemoglobin levels reached 11.8 g/dL with a hematocrit of 39%. The infant's blood group was O Rh‐positive with a negative indirect Coombs test. During the hospitalization in NICU, the newborn underwent electrocardiographic and echocardiographic examinations that resulted normal, whereas neurosongraphy evidenced the presence of choroid plexus cysts at the level of the left caudate thalamus sulcus and bilateral frontal‐parieto‐occipital–periventricular hyperechogenicity at the level of the insula. The newborn was discharged on Day 16, and on follow‐up encephalic ultrasound, no brain damage was found with a normal development at 3 months postnatal follow‐up. Screening tests for congenital infection were all negative, including parvovirus B19. The KB test demonstrated a total of 114 mL of fetal blood within the maternal circulation (normal value < 4 mL), confirming the diagnosis of a spontaneous massive fetal–maternal hemorrhage. Finally, the histological examination of the placenta revealed no anatomical or vascular anomalies.

## 3 Comprehensive Review

**FIGURE 4:**
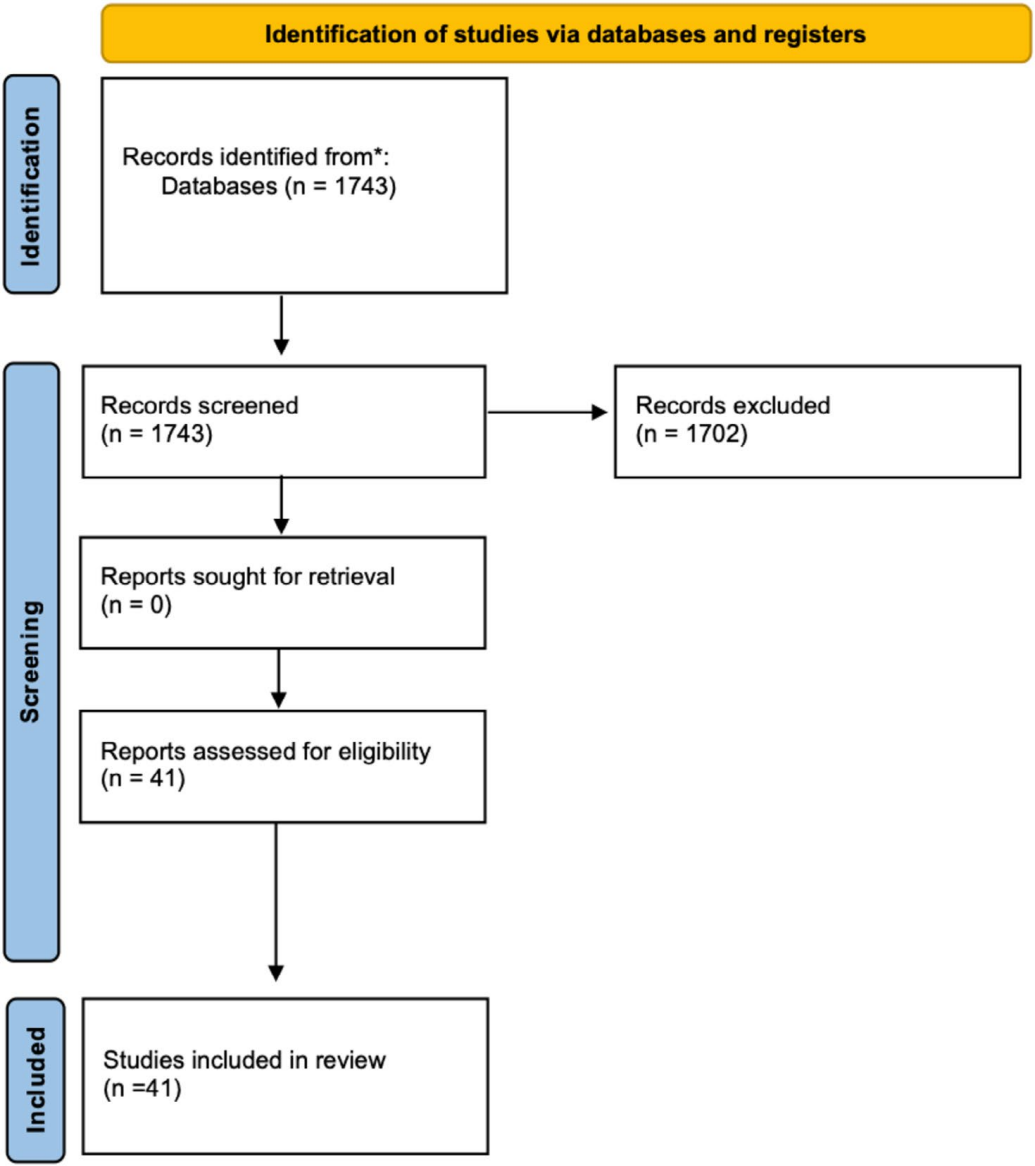
Flow‐chart of included studies.

We performed a comprehensive review of the existing literature from 1966 up to 2023 including 41 articles, with a total of 147 cases of FMH as shown in Figure 4. A search on MEDLINE (PubMed), Google Scholar, and Scopus databases was performed to identify all studies that reported prenatal presentation of fetal–maternal hemorrhage. This review included only articles written in English. Studies were identified using the following keywords: “fetomaternal hemorrhage”; “fetal hemorrhage”; “fetal anemia”; “sinusoidal pattern”; and “reduced fetal movements” in order to have a detailed overview of clinical signs and CTG findings. Following the first literature search, articles were screened by title and abstract in order to filter out studies that did not satisfy the inclusion criteria. After abstracts selection, the remaining full‐text papers were evaluated for eligibility. The inclusion criteria were the clinical description of prenatal fetal–maternal hemorrhages in the third trimester of pregnancy. Exclusion criteria included cases where the fetal–maternal hemorrhage occurred during the first or second trimester of pregnancy; cases where fetal anemia was due to other identifiable causes not related to episodes of fetal‐maternal hemorrhage; articles with missing data; publications after 2023; case reports not written in English.

## 4 Discussion and Analysis of the Results Obtained by the Review

Spontaneous massive FMH is a rare event that can occur at any time during pregnancy, especially during the third trimester. Fetal outcomes depend on the volume of blood lost and whether the loss is acute or chronic. In most cases, the clinical signs are non‐specific and unpredictable. The pathogenesis is often unknown, with 82% being idiopathic, whereas in the remaining 18% it is associated with abdominal trauma, external turning maneuvers, pre‐eclampsia, placental tumors such as choriocarcinoma, or placental abruption (Markham et al. 2006). Studies have shown that regarding fetal and neonatal outcomes, perinatal death occurs in a range of 31%–50% (Kecskes 2003), whereas to a varying extent, infants may develop neurological dysfunction, respiratory distress, persistent pulmonary hypertension, cardiomegaly, and renal failure over time (Giacoia 1997; Stabile et al. 2023).

In the present case described, pregnancy was physiological, without risk factors. The initial mother's report of decreased fetal movements was the first and most important warning sign. Later, the intermittent sinusoidal pattern at CTG for a time of 82 min with an irregular contractile activity associated to a MCA‐PSV of 1.48 MoM, augmented the suspicion of FMH. The newborn's cardiovascular condition with metabolic acidosis at birth and severe anemia (4.3 g/dL) then increased the suspicion of FMH. Finally, the KB test used to demonstrate a total of 114 mL of fetal blood within the maternal circulation confirmed the diagnosis of a spontaneous massive fetal–maternal hemorrhage.

This literature review, based on 41 articles (presented in Tables 1 and 2), has included a total of 147 cases of fetomaternal transfusion and provides a comprehensive analysis of the clinical characteristics, gestational outcomes, and diagnostic and therapeutic implications of this phenomenon.

**TABLE 1:** Number of cases reported in literature, gestational age at presentation, specifying clinical signs, and maternal symptoms.

Author	Number of cases	GA (w)	Clinical signs and maternal symptoms	MCA‐PSV	CTG pattern	Type of delivery	Kleihauer–Betke test	Estimated volume of fetal‐to‐maternal transfusion (mL)
Fischer et al. (1990)	1	31	↓FM	NP	Sinusoidal	CS	Done	180 mL
Heise et al. (1993)	1	33	↓FM	NP	Sinusoidal	CS	Done	195 mL
Tsuda et al. (1995)	6	38 39 41 31 39 40	2 ↓FM	NP	1 sinusoidal 2 reactive 3 NR	5 CS 1 VD	Done	3% 4.2% 4.9% 4.5% 2.9% 3.9%
D'Ercole et al. (1995)	3	26 28 35	FGR Abdominal pain 3 ↓FM	NP	2 NR 1 sinusoidal	NM 2 CS	NM 2 done	NM 180 mL 90 mL
Lipitz et al. (1997)	1	39	NM	NP	Reactive	VD	Done	150–250 mL
Boulos et al. (1998)	9	38 40 38 40 38 38 39 40 39	NM	NP	2 NR 2 reactive 2 sinusoidal	4 CS 5 VD	Done	NM
Bakas et al. (2004)	1	42	↓FM	Normal	Sinusoidal	CS	NP	NM
Salim et al. (2005)	42	> 36	NM	NP	NM	25 CS 17 VD	Done	> 30 mL
Votino et al. (2008)	1	34	↓FM	2 MoM	NR	CS	Done	90 mL
Henningsen et al. (2010)	1	36	Fatigue and palpitations	NP	Preterminal pattern	CS	NM	NM
Watanabe et al. (2010)	1	31	↓FM	NP	Sinusoidal	CS	Done	NM
Ahmed and Abdullatif (2011)	1	35	↓FM	NP	NR	CS	Done	140 mL
Levy‐Zauberman et al. (2011)	1	30	NM	> 1.5 MoM	Sinusoidal	CS	Done	150 mL
Solomonia et al. (2012)	1	28	↓FM	NP	NM	CS	Done	190 mL
Ruffini et al. (2012)	1	37	NM	NP	NM	CS	Done	180 mL
Christensen et al. (2013)	24	29 29 38 32 37 40 34 35 34 38 39 37 40 36 37 40 38 39 39 41 41 38 35 36	13 ↓FM	NP	16 NR 3 sinusoidal 5 reactive	19 CS 5 VD	Done	92–370 mL
Kawano et al. (2013)	1	36	↓FM	Normal	NR	CS	Done	230 mL
Stefanovic et al. (2013)	1	27	↓FM	> 1.55 MoM	Sinusoidal	CS	NM	NM
Singh and Swanson (2014)	1	37	NM	> 1.5 MoM	Recurrent decelerations	CS	Done	75 mL
Kadooka et al. (2014)	18	38 41 37 39 41 37 37 30 39 37 40 37 38 40 38 37 39 30	4 ↓FM	NP	15 NR 2 sinusoidal 4 prolonged bradycardia 3 LOV 2 decelerations variable 12 LD 1 reactive	15 CS 3 VD	NM	NM
Place and Plano (2015)	1	35	↓FM	Abnormal	Sinusoidal	CS	Done	242 mL
Matsui et al. (2015)	1	39	None	NP	NR	CS	Done	140 mL
Umazume et al. (2015)	1	31	Pericardial effusion Persistent nausea, proteinuria	1.76 MoM	NR with LD	CS	NM	NM
Mirza et al. (2015)	1	41	NM	NP	Bradycardia	CS	Done	166–230 mL
Dobrosavljević et al. (2016)	1	39	↓FM weakness, mild fever, joint pains	NP	Sinusoidal	VD	Done	531 mL
Christino Luiz et al. (2019)	1	24	↓FM	2.53 MoM	NR	VD	Done	125 mL
Topping et al. (2019)	1	35	↓FM	> 1.5 MoM	Sinusoidal	CS	Done	268 mL
Stanic et al. 2020	2	34 38	2 ↓FM	Abnormal	1 sinusoidal 1 NR	2 CS	No	NM
Coe (2019)	1	34	↓FM Pericardial effusion	NP	NM	NM	Done	NM
Schmit et al. (2019)	2	38 37	2 ↓FM	NP	2 sinusoidal	2 CS	Done	412 mL 500 mL
Tao et al. (2022)	8	36 37 37 39 37 38 40	2 ↓FM	NM	1 sinusoidal 7 reactive	CS	No	NM
Hookins and Vatsayan (2020)	1	38	↓FM	NP	Sinusoidal	CS	Done	170 mL
Miyahara et al. (2020)	1	27	↓FM	NP	Sinusoidal	CS	Done	986 mL
Gică et al. (2021)	1	37	↓FM	> 1.5 MoM	NR	CS	No	490 mL
Simões et al. (2022)	1	39	None	NP	NR	VD	NM	NM
Signore et al. (2022)	1	40	↓FM	NP	Sinusoidal	CS	Done	146.8 mL
Song et al. (2021)	1	31	Ballantyne syndrome	> 2.0 MoM	Normal	CS	NM	NM
Fortes et al. (2023)	1	39	↓FM	NP	NM	CS	Done	535 mL
Liao et al. (2023)	1	34	↓FM	> 1.5 MoM	Sinusoidal	CS	Done	320 mL
Raymond et al. (2023)	1	36	↓FM	NP	NM	VD	Done	458 mL
Zheng et al. (2023)	2	37 36	2 ↓FM FGR	NP	2 RV Decelerations	CS	Done Done	240 mL NM

*Note:* The peak velocity of systolic blood flow in the middle cerebral artery MCA‐PSV, the CTG pattern, type of delivery, if the Kleihauer–Betke test was performed beats at birth and later the estimated volume of fetal maternal transfusion.

Abbreviations: CS, cesarean section; FGR, fetal growth restriction; FM, reduced fetal movements; LD, late decelerations; LOV, loss of variability; NM, not mentioned; NP, not performed; NR, not reactive; RV, reduced variability; VD, vaginal delivery.

**TABLE 2:** Hemoglobin levels, Apgar score, and pH levels at birth of neonates, whether there was a need for an erythrocyte transfusion at birth, and any long‐term complications.

Author	Number of cases	Level of hemoglobin (new born)	Apgar score (1; 5 min)	pH	RBC required	Long‐term outcomes
Fischer et al. (1990)	1	6.6 g/dL	9/10	NM	96 mL Intrauterine transfusion	Normal
Heise et al. (1993)	1	5.3 g/dL	2/5	7.17	125 mL	Normal
Tsuda et al. (1995)	6	3 g/dL 4 g/dL 4.7 g/dL 2.3 g/dL 2.9 g/dL 7.1 g/dL	7/8 8/8 8/8 2/4 4/6 8/8	NM	NM	NM
D'Ercole et al. (1995)	3	NM	NM 5/7 10/10	NM	None	1 death 2 normal
Lipitz et al. (1997)	1	NM	9/10	NM	Intrauterine transfusion at 27 weeks of 30 mL	Normal
Boulos et al. (1998)	9	7.3 g/dL 10.5 g/dL 4.7 g/dL 5.2 g/dL 9.6 g/dL 8.9 g/dL 8.1 g/dL 6.2 g/dL 3.2 g/dL	10/10 9/10 1/3 5/8 9/10 9/9 8/9 8/9 0/2	NM	5 done 4 none	7 normal 1 psychomotor retardation 1 death
Bakas et al. (2004)	1	8 g/dL	1/5	7.1	Done	NM
Salim et al. (2005)	42	NM	NM	NM	NM	NM
Votino et al. (2008)	1	2.9 g/dL	10/10	NM	120 mL	Normal
Henningsen et al. (2010)	1	2.1 g/dL	0/4	NM	Done	Severe distension of the right side of the heart
Watanabe et al. (2010)	1	3.1 g/dL	4/8	7.25	37 mL	Normal
Ahmed and Abdullatif (2011)	1	3.2 g/dL	2/1	7.06	56 mL	Death
Levy‐Zauberman et al. (2011)	1	7 g/dL	2/NM	7.37	Done	Normal
Solomonia et al. (2012)	1	1.4 g/dL	1/3	6.8	20 mL	Seizures Hydrocephaly Ventriculomegaly
Ruffini et al. (2012)	1	3.8 g/dL	7/8	7.34	59.6 mL	Intraventricular hemorrhage; mild ventriculomegaly
Christensen et al. (2013)	24	1.4 g/dL 1.4 g/dL 1.7 g/dL 2.3 g/dL 2.6 g/dL 2.7 g/dL 2.8 g/dL 3.1 g/dL 3.5 g/dL 3.8 g/dL 4.4 g/dL 4.8 g/dL 5.4 g/dL 5.6 g/dL 5.8 g/dL 5.8 g/dL 6.1 g/dL 6.8 g/dL 7.9 g/dL 8.3 g/dL 8.6 g/dL 9.5 g/dL 9.5 g/dL 10.2 g/dL	0/1 0/0 0/? 1/3 2/5 0/3 4/5 7/8 4/6 8/8 2/3 1/3 4/7 8/8 8/9 8/9 6/8 7/8 1/7 3/9 8/9 6/8 2/2 8/8	7.05 7.15 7.16 7.06 7.18 7.03 7.5 7.23 7.09 7.33 7.04 7.22 7.21 7.25 7.21 7.15 7.35 7.31 7.01 NM 7.24 7.01 7.42 7.32	21 done 3 none	23 normal 1 death
Kawano et al. (2013)	1	4.3 g/dL	5/5	NM	Done	Normal
Stefanovic et al. (2013)	1	6.0 g/dL	3/NM	7.22	75 mL intrauterine transfusion + 20 mL postnatal transfusion	Normal
Singh and Swanson (2014)	1	4 g/dL	1/2	6.95	Done	NM
Kadooka et al. (2014)	18	4.7 g/dL 6.2 g/dL 6.6 g/dL 5.8 g/dL 4.4 g/dL 6.6 g/dL 3.4 g/dL 6.2 g/dL 5.7 g/dL NM 1.8 g/dL 3.9 g/dL 3.1 g/dL 3.4 g/dL 2.7 g/dL 3.9 g/dL 2.2 g/dL 5.0 g/dL 6.1 g/dL	6/9 7/7 4/6 8/8 3/6 4/5 5/8 4/8 0/0 NM 1/3 6/8 5/7 2/2 2/6 2/7 4/6 0/0 4/8	NM 7.02 7.27 7.34 7.27 7.23 7.41 7.39 7.12 NM 6.94 NM 7.24 6.98 7.09 7.12 7.10 7.03 7.22	None	9 normal 1 MR 4 CP 1 CP + MR + epilepsy 1 CP + MR 1 CP + ADHD 1 CP + epilepsy
Place and Plano (2015)	1	2.1 g/dL	1/3	NM	123 mL	Normal
Matsui et al. (2015)	1	6.9 g/dL	2/3	6.81	Done	NM
Umazume et al. (2015)	1	3.6 g/dL	6/8	6.97	50 mL	Normal
Mirza et al. (2015)	1	NM	0/0	7.24	None	Death
Dobrosavljević et al. (2016)	1	5.7 g/dL	1/2	6.8	532	Normal
Christino Luiz et al. (2019)	1	NM	9/8	7.42	Intrauterine transfusion (50 mL)	Normal
Topping et al. (2019)	1	3.2 g/dL	1/4	7.33	47.4 mL	Normal
Stanic et al. 2020	2	3.3 g/dL 5.1 g/dL	4/5 6/8	NM	Done	NM
Coe (2019)	32	NM	NM	NM	None	NM
Schmit et al. (2019)	2	2.7 g/dL 1.7 g/dL	3/7 1/3	7.2 7.22	None	Normal Normal
Tao et al. (2022)	8	2.5 g/dL 3 g/dL 3.7 g/dL 3.9 g/dL 4 g/dL 4.4 g/dL 4.5 g/dL 5.3 g/dL	3/8 8/9 9/10 10/10 9/9 9/9 7/9 9/9	7.16 7.37 7.37 7.41 7.34 7.32 7.27 7.36	NM	Normal
Hookins and Vatsayan (2020)	1	4.7 g/dL	8/7	NM	Done	NM
Miyahara et al. (2020)	1	1.2 g/dL	2/2	7.14	180 mL	Normal
Gică et al. (2021)	1	3.6 g/dL	4/6	7.12	90 mL	Normal
Simões et al. (2022)	1	3.6 g/dL	7/9	7.09	96 mL	Normal
Signore et al. (2022)	1	3.5 g/dL	3/4	7.09	Done	Death
Song et al. (2021)	1	5.8 g/dL	2/5	7.04	70 mL	Normal
Fortes et al. (2023)	1	8.1 g/dL	0/0	NM	None	Death
Liao et al. (2023)	2	2.8 g/dL 2.4 g/dL	2/7 2/7	7.27 7.32	80 mL 72 mL	Normal Normal
Raymond et al. (2023)	1	NM	0/0	NM	None	Death
Zheng et al. (2023)	1	2.6 g/dL 5.2 g/dL	6/7 9/9	7.19 NM	155 mL 70 mL	Normal Normal

Abbreviations: ADHD, attention deficit/hyperactivity disorder; CP, cerebral palsy; MR, mental retardation; NM, not mentioned.

In fact, as shown in Table 1 (Christensen et al. 2013; Watanabe et al. 2010; Votino et al. 2008; Tsuda et al. 1995; Topping et al. 2019; Dobrosavljević et al. 2016; Gică et al. 2021; Tao et al. 2022; Stanic et al. 2020; Schmit et al. 2019; Salim et al. 2005; Bakas et al. 2004; Place and Plano 2015; Heise et al. 1993; Ahmed and Abdullatif 2011; Boulos et al. 1998; Liao et al. 2023; Hookins and Vatsayan 2020; Raymond et al. 2023; Levy‐Zauberman et al. 2011; Matsui et al. 2015; Kawano et al. 2013; Coe 2019; D'Ercole et al. 1995; Christino Luiz et al. 2019; Fischer et al. 1990; Simões et al. 2022; Umazume et al. 2015; Akorsu et al. 2019; Ruffini et al. 2012; Henningsen et al. 2010; Mirza et al. 2015; Singh and Swanson 2014; Stefanovic et al. 2013; Signore et al. 2022; Fortes et al. 2023; Solomonia et al. 2012; Zheng et al. 2023; Lipitz et al. 1997; Kadooka et al. 2014; Miyahara et al. 2020; Song et al. 2021), the data collected indicate that, although fetomaternal transfusion can occur at any time during pregnancy, it is most common at term, with an average gestational age of 36 weeks. However, the observed range is wide, with cases documented as early as 24 weeks and as late as the beginning of the 42nd week of pregnancy. This highlights the importance of close monitoring of patients throughout the third trimester, particularly in high‐risk pregnancies or those complicated by suggestive clinical signs.

Although decreased fetal movements is generally the most common symptom reported among pregnant women (Maier et al. 2015), observed in 51 cases (34.7%), FMH transfusion can also be associated with a variety of other symptoms, such as persistent nausea, abdominal pain, febrile episodes, or elevated proteinuria levels, emphasizing the importance of a multidisciplinary, and vigilant clinical approach to nonspecific symptoms. Interestingly, just one case reported no maternal symptoms, highlighting the potential for asymptomatic presentations and the importance of routine monitoring.

The sinusoidal pattern is the most common CTG finding and is suggestive of fetal anemia (Cozzolino et al. 2017). Other CTG anomalies were described, such as absence of acceleration, presence of recurrent late decelerations, nonspecific decelerative patterns, and finally fetal tachycardia, caused by activation of the fetal compensatory mechanism of secretion of adrenergic‐type catecholamines in response to hypoxic stress, associated with fetal vascular redistribution. In our literature review, 26 cases (17.6%) presented a sinusoidal pattern on cardiotocography. The presence of reactive CTG in only one case, which allowed vaginal delivery, demonstrates the possibility of personalized interventions according to the severity of the fetal condition. These results highlight the need for CTG as a real‐time monitoring tool to help guide decision‐making in cases of suspected fetal compromise.

Additionally, as reported by the studies in Table 1 another important and useful hallmark that plays an important role in assessing and monitoring suspected fetal anemia is the MCA‐PSV (Mari et al. 2000). FMH is generally associated with an MCA‐PSV ≥ 1.5 multiple median (MoM), as it correlates with moderate or severe fetal anemia. Doppler evaluation of fetal MCA‐PSV is based on the principle that MCA‐PSV increases with decreasing fetal hemoglobin levels (Eichbaum et al. 2006). In this study, an increased MCA‐PSV (> 1.5 MoM) was observed in 10 cases (6.8%), which is an important indicator of fetal anemia. Notably, three of these cases presented with values exceeding 2 MoM, indicating severe anemia.

Moreover, as reported in Table 1, the most widely used test for the detection of fetal cells in maternal blood remains the KB test (Boller et al. 2021; Medearis et al. 1984). Other methods reported in the literature are flow cytometry and liquid chromatography. Depending on the obtained result, the fetal blood volume lost into maternal circulation can be estimated according to Mollison's formula: (percentage of fetal erythrocytes × 1800)/100 × 1.22 = mL of fetal blood (Mollison 1972). In this literature review, the KB test, used to estimate the volume of fetal‐to‐maternal hemorrhage, showed a wide range of results, with an average volume of blood of 264 mL, with a range from a minimum of 30 mL to a maximum of 986 mL. This variability reflects the heterogeneity of cases and proves that the extent of hemorrhage may not always correlate with clinical severity or outcomes. It is worth noting that volumes exceeding 80–150 cc are often associated with significant neonatal complications, justifying prompt intervention. Overall, the data underscore the critical role of timely diagnosis and intervention in managing fetal anemia.

Management of massive FMH depends mainly on gestational age at diagnosis. When a 34–36‐week pregnancy is complicated with FMH and there are signs of fetal damage, immediate birth is justified. Correction of anemia should be considered before 32 weeks of gestation. When FMH occurs before 32 weeks, intrauterine transfusion is a well‐established procedure for treating fetal anemia (Lindenburg et al. 2014). However, its absence in other cases suggests either variability in clinical practice or gaps in documentation, which could limit the ability to standardize its use in similar contexts. Of the 141 cases reviewed, cesarean delivery was performed in 68.7% (101 cases), whereas 24.4% (36 cases) resulted in spontaneous vaginal delivery. The preference for cesarean delivery in over two‐thirds of the cases reflects the need for rapid intervention in situations of fetal risk, particularly in the presence of signs of severe anemia or acute fetal distress.

Although Table 1 (Christensen et al. 2013; Watanabe et al. 2010; Votino et al. 2008; Tsuda et al. 1995; Topping et al. 2019; Dobrosavljević et al. 2016; Gică et al. 2021; Tao et al. 2022; Stanic et al. 2020; Schmit et al. 2019; Salim et al. 2005; Bakas et al. 2004; Place and Plano 2015; Heise et al. 1993; Ahmed and Abdullatif 2011; Boulos et al. 1998; Liao et al. 2023; Hookins and Vatsayan 2020; Raymond et al. 2023; Levy‐Zauberman et al. 2011; Matsui et al. 2015; Kawano et al. 2013; Coe 2019; D'Ercole et al. 1995; Christino Luiz et al. 2019; Fischer et al. 1990; Simões et al. 2022; Umazume et al. 2015; Akorsu et al. 2019; Ruffini et al. 2012; Henningsen et al. 2010; Mirza et al. 2015; Singh and Swanson 2014; Stefanovic et al. 2013; Signore et al. 2022; Fortes et al. 2023; Solomonia et al. 2012; Zheng et al. 2023; Lipitz et al. 1997; Kadooka et al. 2014; Miyahara et al. 2020; Song et al. 2021) primarily focuses on the typical signs and symptoms used for the diagnosis of fetomaternal transfusion and its management at term, Table 2 shifts emphasis to the fetal consequences of this condition. It specifically shows important factors such as hemoglobin levels at birth, the Apgar scores, the pH values at delivery, the requirement for a red blood cell transfusion (RBC), and finally long‐term outcomes.

In 98 cases presented in Table 2 (Christensen et al. 2013; Watanabe et al. 2010; Votino et al. 2008; Tsuda et al. 1995; Topping et al. 2019; Dobrosavljević et al. 2016; Gică et al. 2021; Tao et al. 2022; Stanic et al. 2020; Schmit et al. 2019; Salim et al. 2005; Bakas et al. 2004; Place and Plano 2015; Heise et al. 1993; Ahmed and Abdullatif 2011; Boulos et al. 1998; Liao et al. 2023; Hookins and Vatsayan 2020; Raymond et al. 2023; Levy‐Zauberman et al. 2011; Matsui et al. 2015; Kawano et al. 2013; Coe 2019; D'Ercole et al. 1995; Christino Luiz et al. 2019; Fischer et al. 1990; Simões et al. 2022; Umazume et al. 2015; Akorsu et al. 2019; Ruffini et al. 2012; Henningsen et al. 2010; Mirza et al. 2015; Singh and Swanson 2014; Stefanovic et al. 2013; Signore et al. 2022; Fortes et al. 2023; Solomonia et al. 2012; Zheng et al. 2023; Lipitz et al. 1997; Kadooka et al. 2014; Miyahara et al. 2020; Song et al. 2021), the mean hemoglobin level at birth in neonates affected by fetomaternal transfusion was 4.69 g/dL, with a minimum value of 1.2 g/dL and a maximum value of 10.5 g/dL. These findings underscore the severity of anemia in many of these neonates. The mean arterial pH at birth was 7.19, with a lower limit of 6.8 and a maximum value exceeding 7.5, indicating variable degrees of metabolic acidosis associated with cases with a normal acid–base status. In most patients, immediate intervention was required, with red blood cell transfusions performed either at birth or within the first few days of life. This highlights the critical need for rapid neonatal management in cases of significant fetomaternal transfusion. Finally, regarding long‐term outcomes, although most neonates achieved favorable long‐term outcomes, several unfavorable effects were reported. Specifically, 8 cases of neonatal death, 8 neonates with cerebral palsy, 2 cases of ventriculomegaly, 3 patients with mental retardation, and at least 2 cases with epilepsy and one with diagnostic ADHD disorder, further emphasizing the need for long‐term follow‐up in order to identify and manage potential developmental or neurological complications associated with fetomaternal transfusion.

These findings underscore the importance of accurate and quick diagnosis of fetomaternal transfusion, based on a combination of clinical, ultrasonographic, and laboratory investigations. The KB test remains the most used method for quantifying the volume of transferred blood, although it is subject to limitations in sensitivity and specificity. In parallel, monitoring of the middle cerebral artery and CTG remains key tools for early identification of fetal anemia. Future studies should aim to address these gaps by including larger cohorts and ensuring uniform data collection to validate these findings and improve clinical outcomes.

## 5 Conclusions

**FIGURE 5:**
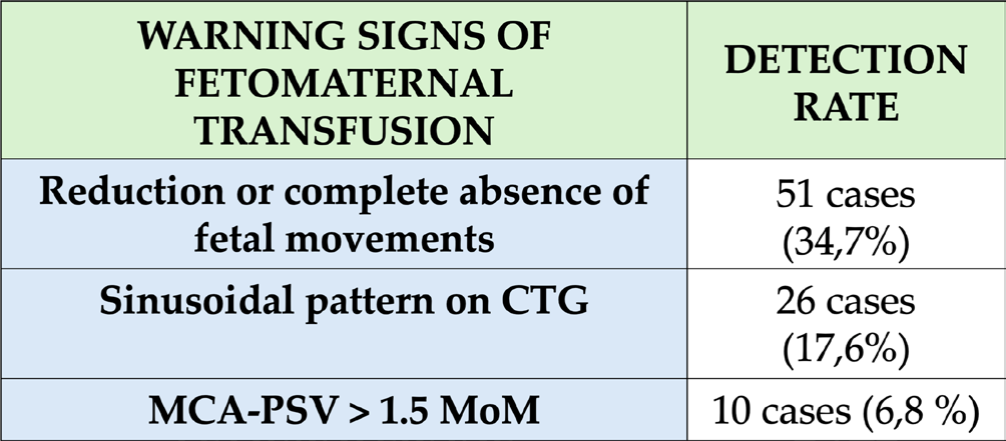
Table showing the most important warning signs of fetomaternal transfusion.

Our study shows that in most cases, the clinical signs of FMH are nonspecific and unpredictable, making timely diagnosis challenging. Indeed, massive acute bleeding can lead to devastating outcomes, including fetal death. The most frequently maternal reported symptom of significant FMH (Figure 5) is a reduction or complete absence of fetal movements, which serves as an important warning sign. On cardiotocography, the findings can vary, often presenting nonspecific deceleration patterns. However, the sinusoidal pattern is the most characteristic finding associated with severe fetal anemia and, consequently, FMH. Doppler ultrasound assessment of the peak systolic velocity of the middle cerebral artery (MCA‐PSV) is a crucial tool for evaluating and monitoring suspected cases of fetal anemia. An MCA‐PSV value equal to or greater than 1.5 multiples of the median (MoM) generally indicates moderate to severe anemia, providing a valuable, noninvasive diagnostic marker. Confirmation of FMH, however, requires the detection of fetal cells in maternal blood, with the KB test remaining the most widely used diagnostic method. The management of massive FMH primarily depends on the gestational age at diagnosis. In pregnancies complicated by FMH between 34 and 36 weeks of gestation, and in the presence of signs of fetal compromise, immediate delivery is often the recommended course of action. For pregnancies diagnosed earlier, particularly before 32 weeks, interventions to correct fetal anemia, such as intrauterine transfusion, should be carefully considered to optimize neonatal outcomes. The aim of this case report, with a review of the literature, was to highlight the most common clinical signs and symptoms associated with the diagnosis of FMH. These findings justify future studies aimed to establish standardized protocols for the investigation and management of suspected cases of FMH, ensuring a more systematic and effective approach to this potentially life‐threatening condition.

## Conflicts of Interest

The authors declare no conflicts of interest.

## Data Availability

The data that support the findings of this study are available from the corresponding author upon reasonable request.
